# PiSCES: Pi(scine) stream community estimation system

**DOI:** 10.1016/j.envsoft.2020.104703

**Published:** 2020-05-01

**Authors:** Mike Cyterski, Craig Barber, Mike Galvin, Rajbir Parmar, John M. Johnston, Deron Smith, Amber Ignatius, Lourdes Prieto, Kurt Wolfe

**Affiliations:** a United States Environmental Protection Agency, National Exposure Research Laboratory, Athens, GA, USA; b Student Services Contractor, United States Environmental Protection Agency, National Exposure Research Laboratory, Athens, GA, USA; c ORISE Research Associate, Oak Ridge Institute for Science and Education, Oak Ridge, TN, USA

**Keywords:** Fish distributions, Community structure, Community size spectra

## Abstract

The Piscine Stream Community Estimation System (PiSCES) provides users with a hypothesized fish community for any stream reach in the conterminous United States using information obtained from Nature Serve, the US Geological Survey (USGS), StreamCat, and the Peterson Field Guide to Freshwater Fishes of North America for over 1000 native and non-native freshwater fish species. PiSCES can filter HUC8-based fish assemblages based on species-specific occurrence models; create a community abundance/biomass distribution by relating relative abundance to mean body weight of each species; and allow users to query its database to see ancillary characteristics of each species (e.g., habitat preferences and maximum size). Future efforts will aim to improve the accuracy of the species distribution database and refine/augment increase the occurrence models. The PiSCES tool is accessible at the EPA’s Quantitative Environmental Domain (QED) website at https://qed.epacdx.net/pisces/

## 1. Introduction

There are over 3.5 million miles of rivers and streams in the United States and many contain fish. Fish abundance and diversity have been used as biological indicators of stream condition (*e.g*., indices of biotic integrity), in addition to macroinvertebrate metrics (e.g., EPT taxa) and standard water quality measurements (*e.g*., DO, pH, conductivity) (Davis et al., 1996; Hughes et al., 1999; Karr, 1981; Karr and Dudley, 1981; Karr et al., 1986; Miller et al., 1988; Simon et al., 1995; Hill et al., 2017; Carlisle et al., 2009; Maloney et al., 2009). Because fish are longer lived than aquatic macroinvertebrates, they integrate a wide array of stream and watershed factors related to the quality of lotic systems on the order of years (Plafkin et al., 1989; Karr and Chu, 1999; Wesche et al., 1999; Kovacs et al., 2002). Fish species are grouped into tolerant and intolerant categories based on known habitat affinities, with integrity indices developed for use within states and regions of interest (Fausch et al., 1990; Simon, 1999; Stepenuck et al., 2002; Stoddard et al., 2006).

Given that a fish community reflects on the state of the system it occupies, government agencies (federal, state, local), non-governmental groups and academic researchers routinely conduct field sampling to ascertain what fishes are present (Weaver and Garman, 1994; Yoder et al., 1998; Angermeier and Winston, 1999; Barbour et al., 1999; Wang et al., 2000; USEPA, 2013). Sampling methods vary, but some of the most popular are backpack electrofishing (multiple pass and depletion), seining (electric and otherwise) and boat shocking.

Many factors determine where fish species are found, including physical stream habitat (e.g., flow, depth, woody debris presence and substrate quality), ecological interactions (including introduced and invasive species), levels of stressors (sedimentation, pH, temperature, nutrients) and historical biogeography (Fausch et al., 1990; Simon, 1999; Jackson et al., 2001; Quist et al., 2004; Rashleigh et al., 2005; Bertolo and Magnan, 2006; Kennard et al., 2007; Dauwalter et al., 2008; Pool et al., 2010; Baxter and Hauer, 2000). Fish distributions are not static but change through time as functions of life history, disturbance, seasonal and prevailing hydrologic regimes, species introductions, climate change and food availability (Shuter and Post, 1990; Taylor and Warren, 2001; Rahel, 2002; Taylor et al., 2006, 2008bib_Taylor_et_al_2006bib_Taylor_-et_al_2008; Schaefer et al., 2012; Erős et al., 2014). Field sampling, however, can be time consuming, expensive, biased by gear selectivity and compromised by the dynamic nature of fish assemblages in lotic systems. Alternative methods of estimating fish species and abundance are needed to augment field sampling efforts, and a variety of models have been developed for this purpose (Oakes et al., 2005; Fransen et al., 2006; McCleary and Hassan, 2008). An empirical modeling approach derived from field data would have wide geographic application and utility for stream condition assessment.

Multivariate statistical methods have been used to relate fish distributions to environmental characteristics using a variety of analytical techniques (Angermeier and Winston, 1999; Kelso and Johnson, 1991; Madejczyk et al., 1998; Ornellas and Coutinho, 1998; Saiki and Martin, 2001; Kendrick and Francis, 2002; Schweizer and Jager, 2011; Troia and McManamay, 2020; Brenden et al., 2008; Leathwick et al., 2011). Stream sampling data collected by the US Environmental Protection Agency (USEPA) in the Mid-Atlantic Highlands region of the eastern US (Herlihy et al., 2000) was used for a k-means cluster analysis of relative abundance data, followed by a discriminant analysis to predict a stream’s potential fish assemblage based on stream and watershed characteristics (Cyterski and Barber, 2006). McCormick, Peck (McCormick et al., 2000) found that taxonomic grouping of these data (based on cluster analysis) had higher classification strength than groupings based on geographic (catchment/ecoregion) or stream order. These methods and data have also been used to develop a decision support tool for fisheries management and stream habitat restoration [WHAT-IF, USEPA (USEPA, 2006)]. However, fish community data are not available in all locations, and not always internet-accessible even when present. Data availability, accessibility and quality are therefore barriers to the widespread use of fish as biological indicators.

To overcome the reliance on field data and the resources needed to compile and interpret fish sampling databases, an alternative approach to stream fish assemblage modeling was developed: The Piscine Stream Community Estimation System, or PiSCES. PiSCES is a tool for determining credible fish communities for streams and rivers across the conterminous US. Historic and current range data for over 1000 native and non-native freshwater fish species were combined with information on species characteristics and habitat preferences [NatureServe (2010); Page and Burr (2011); FishBase.Org]. PiSCES incorporates the results of analyses conducted on stream and watershed characteristics to determine species-specific probabilistic occurrence models, which are used to modify presence/absence information based simply on the geographic distributions of each species.

The motivation to develop PiSCES was based in the lack of publicly available fish community data for many US lotic, freshwater habitats, because field sampling is cost-prohibitive for all potential streams and rivers of interest. Fisheries data (i.e., fish species enumeration with accompanying abundance, length and weight measurements) are also not typically available online. In the absence of field data, modeling conducted to evaluate changes in ecosystem services, such as the impact of multiple stressors (e.g., landcover change, riparian buffer zone creation and corresponding changes in suspended and dissolved inputs) on fish community provisioning also requires one to specify a fish community as an initial condition for a stream or river of interest. Therefore, a reliable fish community dataset is essential to accurately forecast fish community biomass. This includes integrated modeling approaches in which ecological models [*e.g*., BASS (Barber, 2012) and HSI (Rashleigh et al., 2005)] are coupled with hydrologic and water quality models (Johnston et al., 2011), but that are limited in their transferability to other watersheds, including nearby freshwater systems with differing habitats and fish autecology. PiSCES solves this problem by providing a reliable estimate of the fish community for lotic systems throughout the CONUS, while also accounting for habitat suitability constraints on fish species. Development of PiSCES enhanced the transferability of an integrated ecological modeling system in a prior study (Johnston et al., 2017). PiSCES also improves the ability to conduct cumulative impact assessment under the National Environmental Policy Act (NEPA, http://www.epa.gov/compliance/nepa/index.html) and other regulatory frameworks (e.g., Clean Water Act, Total Maximum Daily Load). In developing PiSCES, we strove to combine the best available geographic data on fish distributions with species-specific occurrence models, and to package this information in a user-friendly, web-based software tool. PiSCES can be used for research purposes in support of ecological modeling (*e.g*., Johnston et al. (2017)) or to provide information to an interested citizen-scientist curious about what fish species may be present in their local stream or river.

## 2. Software description

The PiSCES core is written in the Python programming language. The fish properties and distributional ranges (based on 8-digit hydrologic basins, heretofore “HUC8”) are stored in a PostGIS relational database. The software is deployed as a web application using the Django framework for server-side processing. The graphical user interface (GUI) is built using HTML, CSS and Javascript. The PiSCES core functionality is exposed through a collection of RESTful web services that can be accessed independently of the browser-based GUI.

The PiSCES GUI contains three separate tabs:

- Distribution Explorer
- Assemblage Predictor
- Database Explorer

### 2.1. Distribution Explorer

**Fig. 1.**
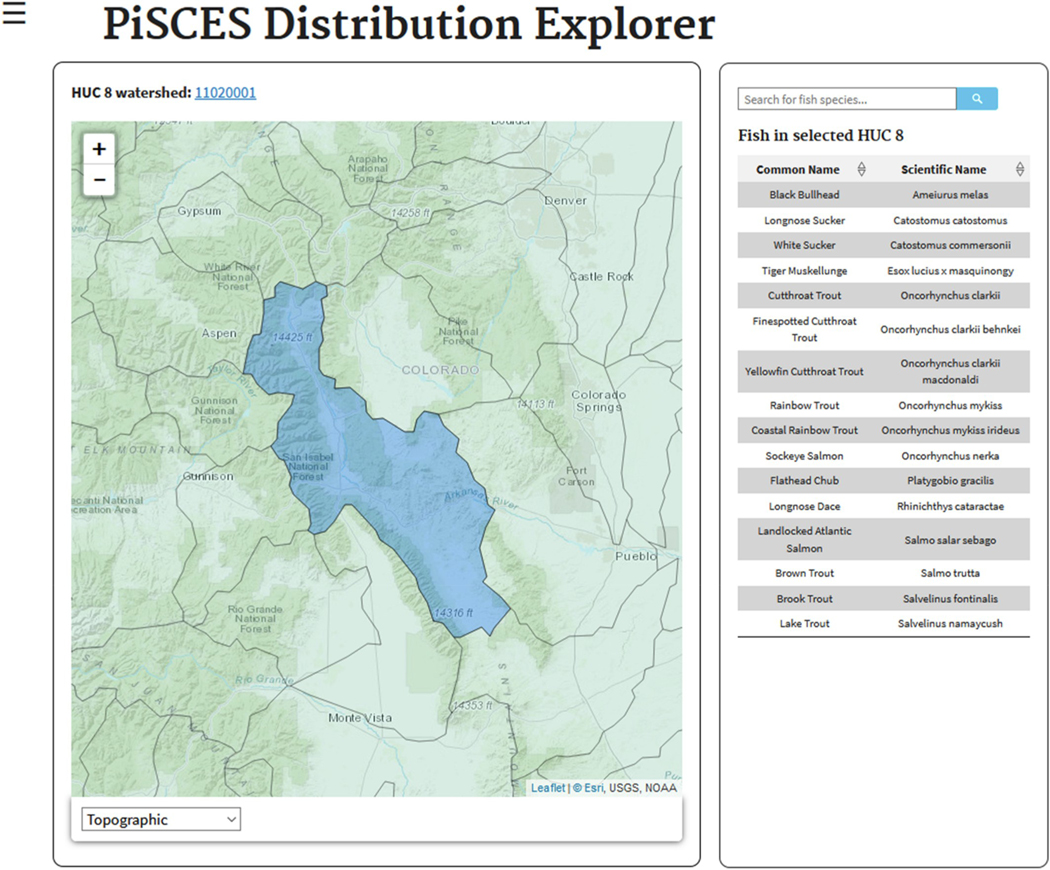
The PiSCES “Distribution Explorer” window with a list of fish species found in the selected HUC8.

Here the user can access a navigable map of the US, where they can pan, zoom and select a HUC8 (defined by the Watershed Boundary Dataset obtained from the USGS at http://nhd.usgs.gov/wbd.html) to see what species are expected in that HUC8, given their known ranges (Fig. 1). A link at the top of the page will redirect the user to the USGS Water Resources site for the watershed.

Users can also search the PiSCES species database by common or scientific name. Clicking on a species in the list to the right of the map will display the currently known geographic distribution for that species, as well as provide species characteristics in a table below. Links to a Google image search and the Wikipedia page for the selected species can also be found at the bottom of the page.

### 2.2. Assemblage Predictor

**Fig. 2.**
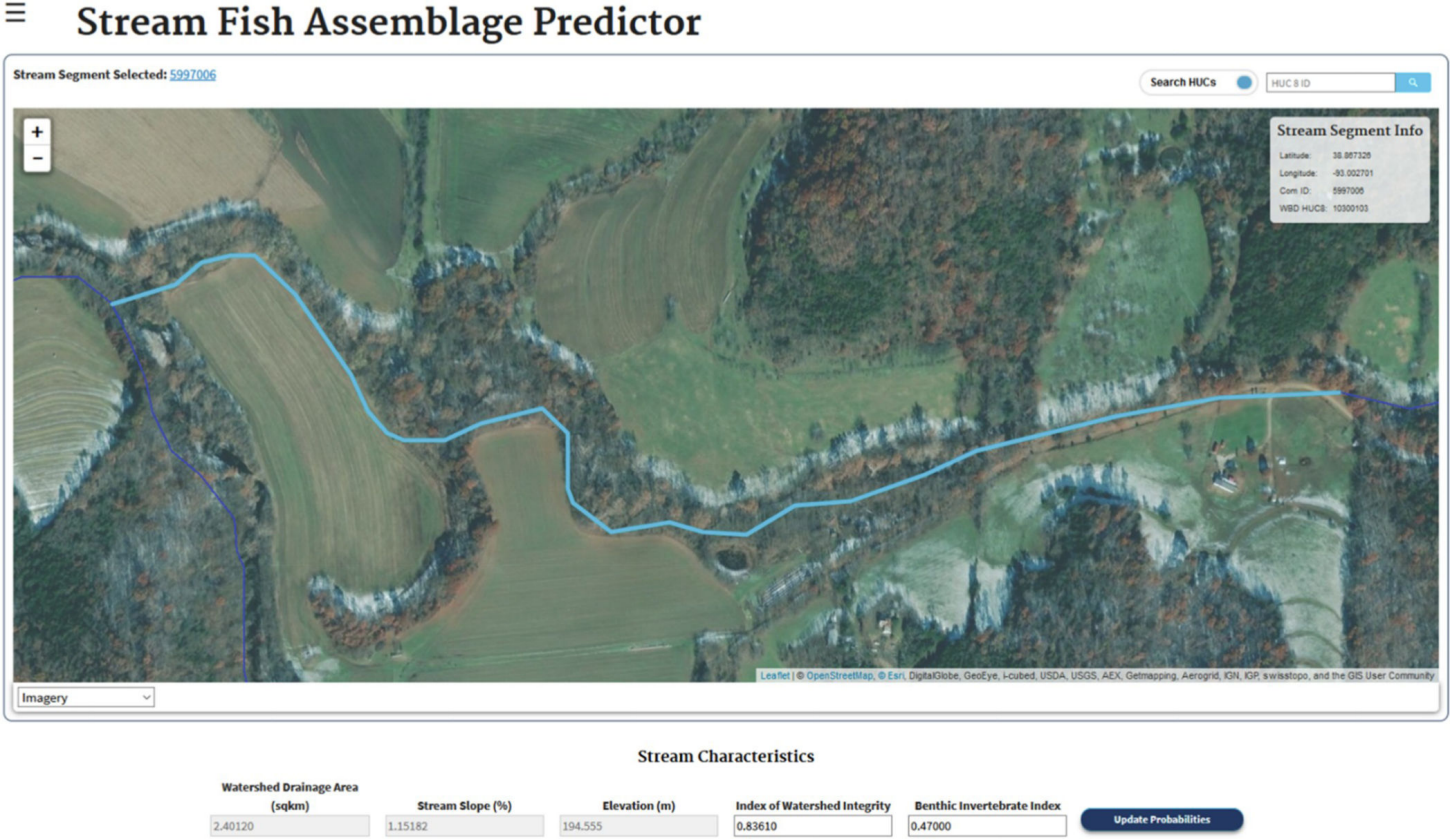
The PiSCES “Assemblage Predictor” showing a highlighted stream segment and the potential filters used to turn the initial HUC assemblage into a more plausible fish community for the selected segment.

A map depicting the 1:100,000 scale stream segments of the National Hydrography Dataset version 2 (NHDPlusV2, McKay, Bondelid (McKay et al., 2012)) for the entire US are displayed (Fig. 2); note that the map must be zoomed to see these individual stream segments. Satellite imagery and map features can assist in stream identification. Once a user clicks an individual stream segment, the entire fish assemblage for that HUC8 is shown in a table on the bottom left of the page. Above the map is a link to the USEPA’s Watershed Report that provides basin characteristics for the chosen segment. Below the map are values for five characteristics of the chosen stream segment which are used to calculate occurrence models to estimate a more plausible fish community for the given stream segment: drainage area (km^2^), stream slope (%), mean catchment elevation (m), the index of watershed integrity (IWI), and the probability of the segment having a good benthic invertebrate multimetric index (BMMI).

These covariates are taken from the StreamCat (Hill et al., 2016) and NHDPlusV2 databases. The IWI attempts to summarize, in a multimetric index, the capacity of a watershed to support a full range of ecological processes and functions necessary for sustaining biotic integrity (Thornbrugh et al., 2018). It assesses the status of six watershed functions: hydrologic regulation, water chemistry, sediment, hydrologic connectivity, temperature and habitat provisioning. The probability of the stream segment having a “good” community of benthic invertebrates is predicted by a random forest model (model development described in (Hill et al., 2017)). The user may change the IWI and the BMMI values (each can vary from 0 to 1) for scenario investigations. In addition, any species can be manually excluded or included from the community by clicking/unclicking the checkbox to the left of the species name.

**Fig. 3.**
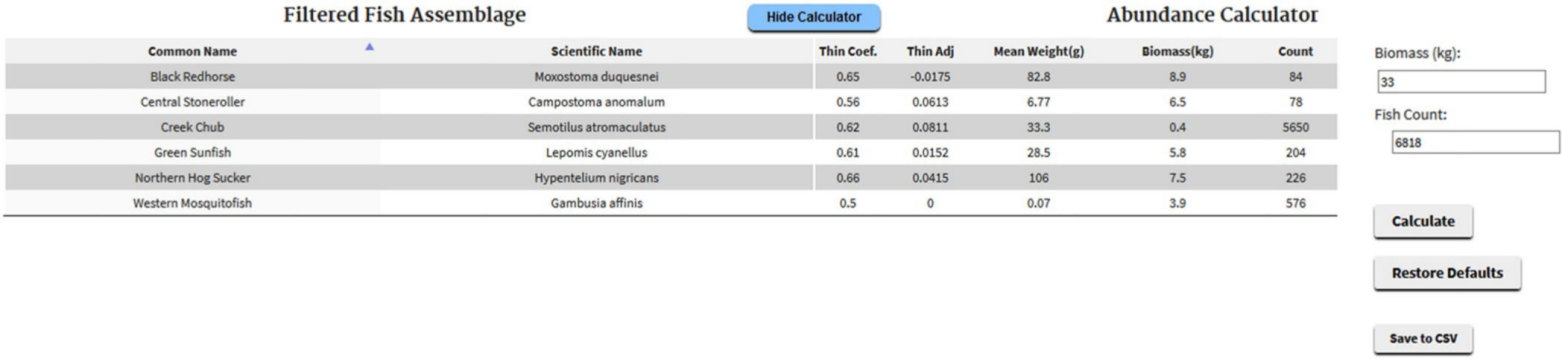
The PiSCES “Assemblage Predictor” showing the abundance/biomass calculations for a fish community.

Under the parameter values is a drop-down menu (“Threshold Filter”) used to set the cutoff for assessing the probability of species-specific occurrence models, which shall be discussed later. There is also a button (“Display/Hide Calculator”) which can be toggled to show/hide the community abundance/biomass calculator (Fig. 3), which will estimate the distribution of total biomass or number of fish amongst the community members.

**Fig. 4.**
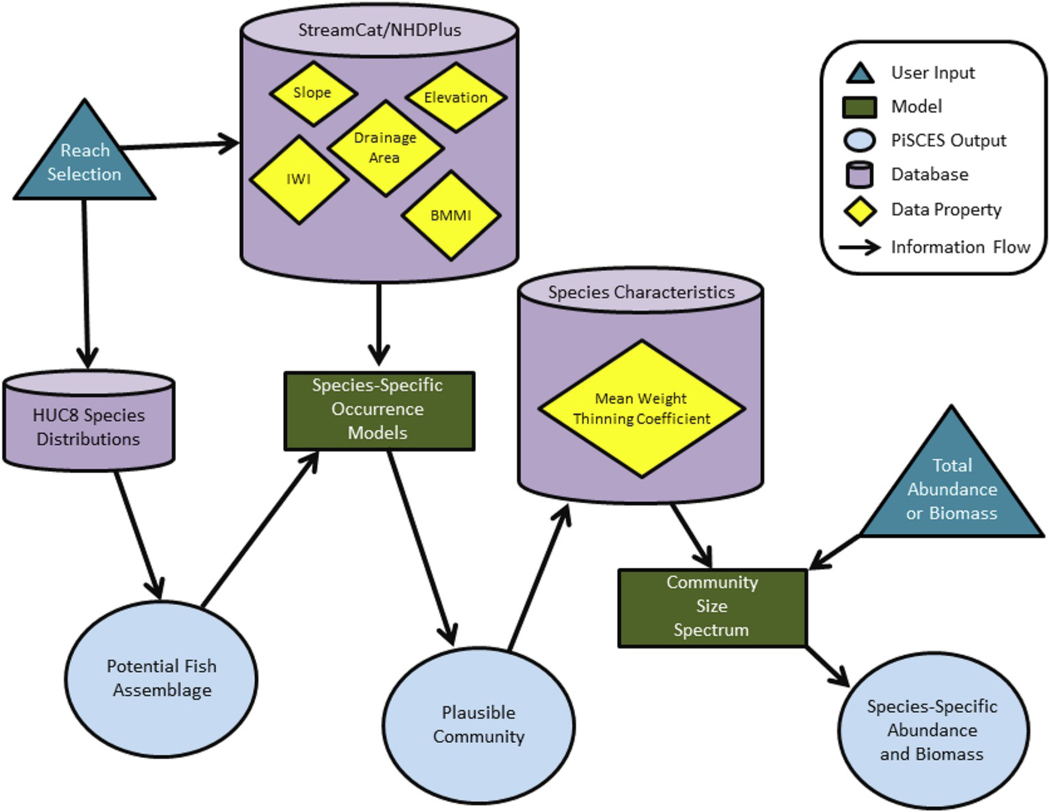
Schematic depiction of the models, user inputs, databases and information flow within the PiSCES Assemblage Predictor.

The Assemblage Predictor output depends on several databases, model calculations and user input. Fig. 4 shows a schematic of how, beginning with a user’s choice of a stream reach (dark blue triangle at the upper left), various pieces of information come together to produce a prediction of numbers or biomass of a fish community for that stream (light blue circle at bottom right). These components will be discussed later.

### 2.3. Database Explorer

**Fig. 5.**
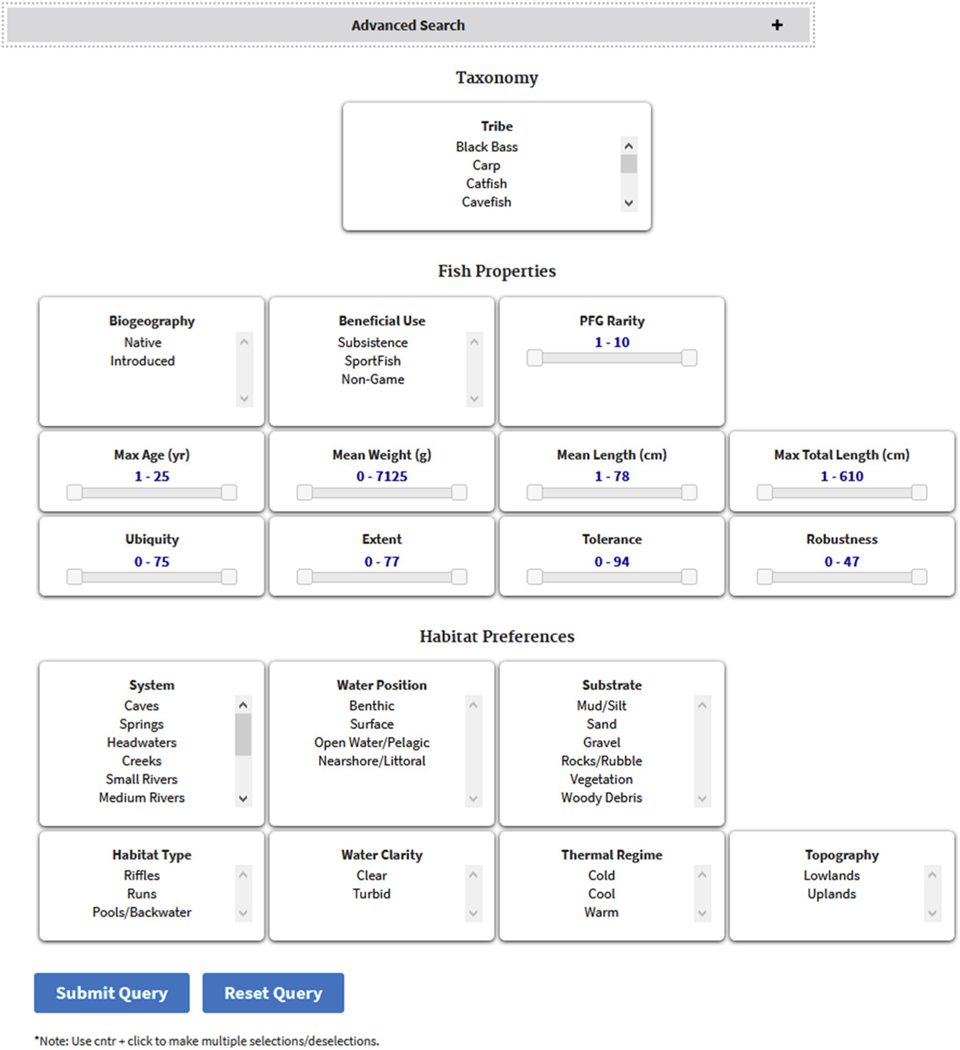
The PiSCES “Database Explorer” window showing the variety of species characteristics that can be used to query the database.

This page allows a user to interact with the PiSCES species database via a query builder that can be manipulated to reveal all species that fit a chosen profile (Fig. 5), such as those having some level of rarity, attaining some maximum body size, or those species that are benthic and prefer riffles.

The list of species that match the query parameters are shown below the selection boxes once a query is submitted. Clicking on a species in this list brings up a table with species characteristics, as well as Google image search and Wikipedia links for that species.

## 3. Scientific foundations

Scientific and common names for fish species were adopted from Page, Espinosa-Perez ( Page et al., 2013). Stream segmentation for the conterminous US was derived from the NHDPlusV2 dataset. In addition, the following information for each fish species was obtained from the Peterson Field Guide to North American Fishes (Page and Burr, 2011), the online NatureServe Explorer (http://explorer.natureserve.org/) and FishBase (http://www.fishbase.org/):

- The rarity of each species inside its range
- The maximum size (body length) that each species attains
- Assorted habitat preferences for each species

### 3.1. Fish distributions

The basis of PiSCES fish assemblage predictions are known current geographic distributions of fish species, which were obtained primarily from two sources:

- Shapefiles of HUC8-based species distributions obtained from NatureServe (2010)
- HUC8-based records of species introductions for native and non-native fishes; from the USGS Nonindigenous Aquatic Species Program: nas.er.usgs.gov/taxgroup/fish/default.aspx

USGS and NatureServe personnel communicate to keep species distributions (primarily those introduced outside of their native ranges) up to date. Larry Page (Florida Museum of Natural History) provided shapefiles for the polygons that appear in the Peterson Field Guide (PFG). These polygons are imprecise at the boundaries of species distributions; whenever possible we chose to use the NatureServe/USGS data. The PFG, however, provided distributions for the following 16 species/sub-species not found in the NatureServe or USGS databases:

**Table:** 

Campostoma spadiceum	Highland Stoneroller
*Cottus hubbsi*	Columbia Sculpin
*Erimyzon claviformis*	Western Creek Chubsucker
*Etheostoma atripinne*	Cumberland Snubnose Darter
*Etheostoma erythrozonum*	Meramec Saddled Darter
*Etheostoma occidentale*	Westrim Darter
*Etheostoma orientale*	Eastrim Darter
*Etheostoma planasaxatile*	Duck Darter
*Etheostoma spilotum*	Cumberland Plateau Darter
*Etheostoma tennesseense*	Tennessee Darter
*Lepomis peltastes*	Northern Sunfish
*Oncorhynchus mykiss newberrii*	Great Basin Rainbow Trout
*Oncorhynchus mykiss gilberti*	Kern Rainbow Trout
*Oncorhynchus mykiss stonei*	Sacramento Rainbow Trout
*Oncorhynchus clarkii macdonaldi*	Yellowfin Cutthroat Trout
*Percina apristis*	Guadalupe Darter

For these fish, the PFG polygons were used. To convert these polygons, which were constructed by drawing a boundary around locations where species have been captured, to hydrologically-based areas (to increase distributional precision), we examined the overlap between the polygons and the map of HUC8’s, considering watershed drainage patterns to determine what basins should be defined as probable locations for the species of interest. HUC8’s with at least 50% of their area inside the PFG distributional polygon were considered to contain that species. We also included HUC8’s in a species’ distribution with overlap greater than 0%, but less than 50%, if the HUC8 was directly upstream or downstream of a HUC8 with greater than 50% overlap. When a distributional polygon was relatively small (*i.e*., intersecting or wholly contained within only a few HUC8’s), then each HUC8 intersecting the polygon was included in the species’ distribution.

### 3.2. Species rarity

As noted in the PFG, rarity is not synonymous with the spatial extent of a species’ range. A species can be very abundant within a tiny range, like a few pools or springs in the case of certain desert pupfish, or a species can be uncommon/rare, and yet have a widespread distribution across a large geographic expanse. For our purposes, rarity relates to how likely a species would be found at a suitable location within its range. We converted the rarity descriptor in the PFG into a numeric scale:

**Table:** 

1: Abundant	6: Uncommon
2: Abundant/Common	7: Uncommon/Rare
3: Common	8: Rare
4: Fairly Common	9: Extremely Rare
5: Common/Uncommon	10: Extinct

A PFG rarity of 10 indicates the species was historically seen in the HUC8 but is currently extinct.

### 3.3. Probabilistic occurrence models

We compiled a dataset of presence/absence fish survey information from a variety of sources:

- USGS Biodata Retrieval System: (https://aquatic.biodata.usgs.gov/landing.action). USGS stream surveys conducted from 1993 to 2017.
- The Multistate Aquatic Resources Information System (MARIS): (https://www.sciencebase.gov/catalog/item/54998234e4b08b255be64e6e). Surveys conducted by state agencies during 1990–2014.
- USEPA National Rivers and Streams Assessment (NRSA): (https://www.epa.gov/national-aquatic-resource-surveys/nrsa). USEPA surveys conducted during 2008–2009.
- USEPA mid-Atlantic Highlands Assessment (EMAP-MAHA): (https://archive.epa.gov/emap/archive-emap/web/html/index-167.html). Records span 1993–1996.
- USEPA Western Assessment (EMAP-WEST): (https://archive.epa.gov/emap/archive-emap/web/html/wstream.html). Records span 1999–2000 and 2002–2006.

In total, this dataset represented 39,073 surveys done on freshwater rivers and streams across the contiguous US (Fig. 6a). For model development, we used the following covariates, available from the NHDPlusV2 and StreamCat databases for each survey site, and described earlier in Section 2.2: drainage area (km^2^), mean catchment elevation (m), stream slope (%), the index of watershed integrity (IWI), and the probability of the segment having a good benthic invertebrate multimetric index (BMMI).

Species-specific probabilistic occurrence models were developed using a generalized boosting method in Python and the “XGBoost” package (Chen and Guestrin, 2016). Models were initially trained using data from 75% of the sites, with 25% of the sites randomly withheld for validation purposes (Fig. 6b).

The process for developing species-specific occurrence models was as follows:

1. Identify all fish species caught in at least 25 surveys within the training dataset (432 species out of a total of 564 species).
2. For each species, create a dataset of survey sites where it was captured, and survey sites within HUC8s where the species occurs, but was not captured.
3. For these sites, obtain the five covariate stream parameters from the StreamCat database (stream slope from the NHDPlusV2 database).
4. Run the XGBoost package (using the XGBClassifier function for a binary response variable) to create a species-specific model of the probability of occurrence for each of the 432 species.

The 432 species represent over 99% of the species by survey site combinations in the training dataset (301,400/303,229). After calculating validation metrics based on applying the fitted models to the withheld testing sites, the testing data were combined with the training data, and new occurrence models were developed for the final PiSCES deployment for those species seen in at least 25 surveys in the combined dataset (n = 449).

The XGBoost package has an array of model parameters that can affect the fitting process and efficacy of the eventual solution. Below are the values we used for developing these models.

- n_estimators: 500
- learning_rate: 0.05
- subsample: 0.75
- min_samples_leaf: 3
- max_depth: 3
- n_iter_no_change: 10

For more details of the effect of these parameters on the fitting algorithm, see the XGBoost documentation (https://xgboost.readthedocs.io/en/latest/python/). The maximum number of trees in a solution (n_estimators) was set to 500; this number produced satisfactory results for all species. The “subsample” fraction (0.75) results in each successive tree in the iterative algorithm being fit to a random 75% of the observations in the training data, which mitigates overfitting. max_depth = 3 means that two and three-way interactions of model covariates could be captured by the model. The min_samples_leaf parameter prevents the model from being unduly influenced by outliers/odd samples. Smaller values of the learning_rate can increase model accuracy, but at the cost of increased computational time and adding trees to the eventual solution. We found a value of 0.05 to work well for these data, as measured by quick convergence to a solution without exceeding the maximum number of allowable estimators. As more trees are added to the solution, the training data error will continue to decline; the validation error (assessed using 10-fold cross validation) initially declines, but then rises if too many trees are used, i.e., the model becomes overfit. The n_iter_-no_change option will stop the iterative solution process if the validation error is not improved over the specified number of iterations.

### 3.4. Fish abundance

Once the initial stream fish assemblage (based simply on HUC8 distributional information) is reduced to a more plausible community (a collection of species that could likely be found in a certain stream given its characteristics), a user may want to estimate the abundance or biomass of individuals of each species in the stream reach, which can be done on the “Assemblage Predictor” tab. For the integrated modeling system and the fish community simulation model that PiSCES was developed to support (Johnston et al., 2011, 2017), this was a necessary initial condition to estimate. PiSCES uses several pieces of information to perform this task. One is the user-specified total abundance or total biomass of fish in the stream reach. For context, we note that in an extensive analysis of stream fish collection data, Barber et al. (2015) found that mean total density of fish was about 7150 fish/ha and mean total fish biomass was about 45 kg/ha, but the variability of these estimates was very large.

A second piece of information is the general negative relationship between the body size of an organism and its abundance in a community and/or population (Sheldon et al., 1972; Pope et al., 1982; Han and Straškraba, 1998; Boicourt et al., 2004; White et al., 2007; McGill, 2008; McGarvey et al., 2010):

Equation 1 $$\;Abundance\;\propto {\left(Mean\;Weight\;,g\right)}^{-\beta }$$

PiSCES uses a species-specific β (thinning exponent) based on the mean weight of a species, as the thinning intensity is also generally size-dependent (McGarvey et al., 2010). PiSCES sets a lower limit for β at 0.5 for very small species (≤ 1g) and an upper limit of 0.75 for the largest species (≥ 1000g). These are considered reasonable values for community-level thinning exponents (Bohlin et al., 1994; Carbone and Gittleman, 2002; Rincón and Lobón-Cerviá, 2002; Savage et al., 2004). For species between 1 and 1000g mean weight, the following equation is used to determine β:

Equation 2 $$\beta =0.5*\text{ (}Mean\;Weight\text{, }g{)}^{0.06}$$

Smaller β values in Equation (2) lead to a flatter size spectrum, where the abundance of larger individuals does not drop off as steeply relative to the abundance of smaller individuals. Larger β values produce a steeper decline in abundance with increasing size, as is often the case in heavily exploited fisheries (Duplisea and Castonguay, 2006). The user can change the default β value for any species, but we suggest staying within the 0.5–0.75 range. As the number of larger fish is already being curtailed by the inverse abundance/weight relationship described by Equation (1), making the impact of larger weight on β too severe (Equation (2)) leads to very small relative biomass of the largest species in a community, which does not agree with the general findings of Barber et al. (2015).

The PiSCES database contains the maximum age, mean weight and mean length for many species based on the data analyses of Barber et al. (2015). For those species not addressed by that study, parameter values were based on regressions of these three parameters versus maximum length (which we had for all species, primarily from the Peterson Guide). The derived regression equations and their R^2^ values:

Equation 3 $$\text{ Mean Species Weight }(\text{g})=0.00654^{*}{\left(Max\;Species\;Length,\;cm\right)}^{2.1675}\;{\text{R}}^{2}=0.74$$

Equation 4 $$\text{Mean Species Length}\;(\text{cm})=4.7*{\left(Mean\;Species\;Weight,\;g\right)}^{0.3184}\;{\text{R}}^{2}=0.96$$

Equation 5a $$\text{ Max Species Age }(\text{yr})=1.333*{\left(Max\;Species\;Length\;,cm\right)}^{0.428}\;{\text{R}}^{2}=0.37$$

The PiSCES database also provides (from Barber et al. (2015)) species-specific values of the coefficients in a power function for weight (g, wet weight) versus length (cm) (*W* = a*L^*b*^). For those species for which these values were not given by Barber et al. (2015), we used the average coefficient across the dataset. For *a* and *b*, these were 0.01135 and 3.07. PiSCES provides a calculator on the Distribution Explorer for converting length to weight and vice versa for any species.

There is undoubtedly variability in the demographics of fish populations in different streams (growth and mortality affecting the mean body weight of a species) depending on the quality of the habitat they occupy, density of competitors and predators, etc. The PiSCES GUI allows the user to modify default values of mean species weight for investigative purposes, e.g., if the user has better information on the mean weight of a species in a specific lotic system or wants to examine what a hypothetical rise or decrease in mean species weight would mean for a species within a community. Within PiSCES, this is close to a zero-sum game, as total community biomass doesn’t increase/decrease greatly if the mean weight of a species is increased/decreased. Instead, the abundance of that species declines/increases to compensate for the weight gain/loss. Because community abundance is derived from relative abundance, the number of individuals in the other species of the community also rise/fall to a lesser extent to compensate for the weight change in a species. In an absolute sense, the numbers of the smallest species change much more than the numbers of the largest fish, but the changes should be similar on a percentage basis.

Table 1 shows how PiSCES would handle a hypothetical community of seven fish species ranging in mean weight from 0.1 to 200g (*e.g*., small cyprinids to largemouth bass), given a total community abundance of 10,000 fish.

Column two provides the weight-based thinning exponent (Equation (2)). Column three is weight-based species abundance (Equation (1)), but these values are difficult to interpret because magnitudes are dependent on the units of weight and choice of β. The relative species abundance in column four is more useful (column three values divided by the sum of column three). Relative abundance is multiplied by total abundance to provide an estimate of the number of individuals of each species (column five). Column six shows the total biomass of each species (N*Mean Weight).

Table 1 conforms with conditions seen in most stream fish communities, *i.e*., the smallest species typically dominate relative abundance (as do the smallest individuals in a population), but their total biomass is relatively small. Larger species have more relative biomass, but there are far fewer of them. If the user specifies a total biomass for the community, PiSCES will find the total abundance that produces the desired community biomass. For example, the community in Table 1 has a total biomass of ≈32 kg. If the user had specified a different total biomass, PiSCES would use the results of an initial attempt as a starting point for adjusting total abundance to attain the desired total biomass.

A completely size-based approach to community estimation is obviously over-simplified, ignoring competitive fitness and environmental tolerance of each species, but the PiSCES abundance/biomass calculator is meant as a first-pass approximation of a community spectrum. Estimates should give a logarithmic sense of species’ abundance; i. e., would the species occur in the tens, hundreds, or thousands of individuals. If a user wants to simulate the abundance of a particularly hardy, tolerant species, they could decrease its weight-based thinning exponent. In the same manner, a user might want to increase the thinning exponent for a sensitive, intolerant species under adverse conditions.

**Fig. 7.**
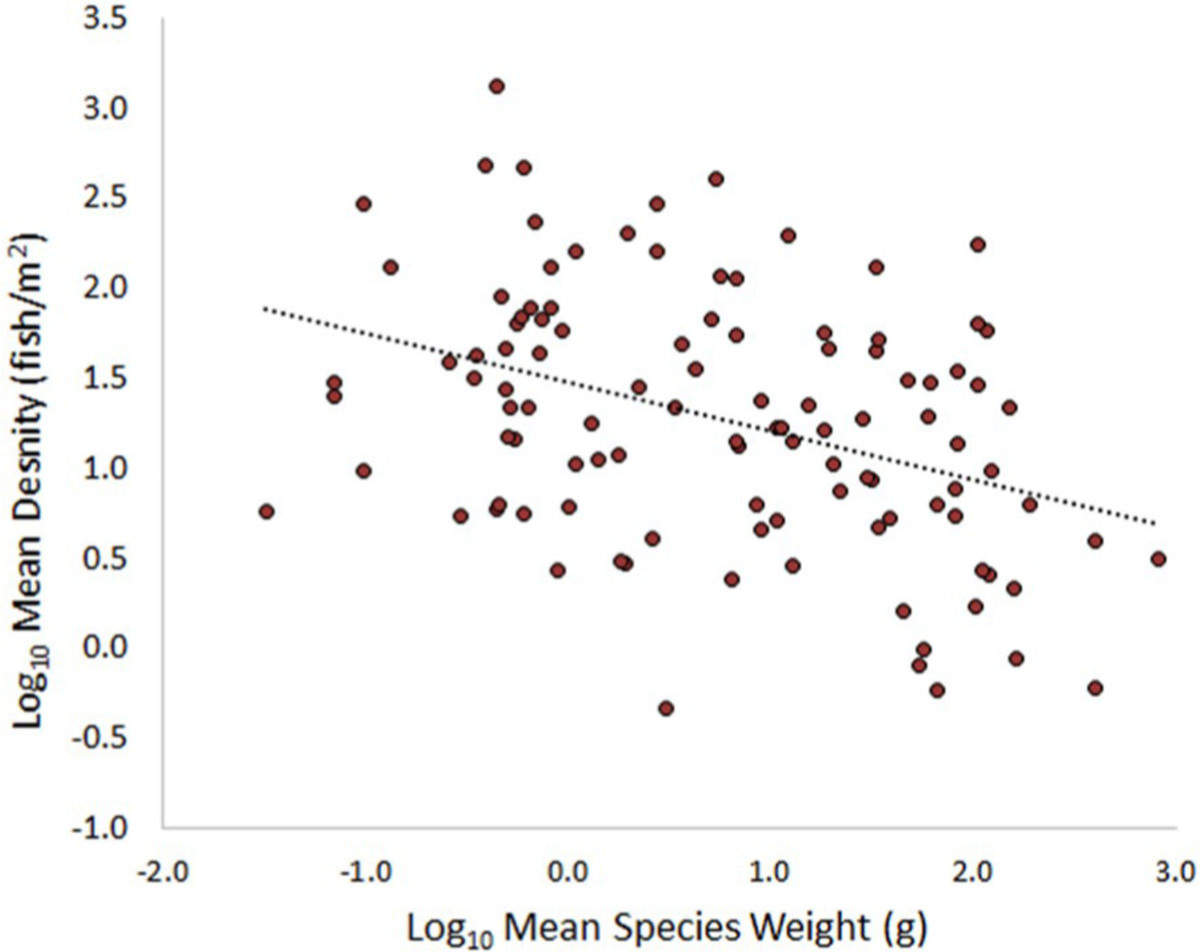
Negative linear relationship between mean density (fish/m2) and mean species weight (g) for 108 species from data in Barber et al. (2015). The mean residual value is zero, and the standard deviation of the residuals was 0.65.

We found a negative relationship (Fig. 7) between mean species weight (g) and mean density (fish/m^2^) for 108 species found in 8 or more sample surveys using the data presented in Barber et al. (2015). Clearly, more factors than mean species weight are important for determining a species’ density. However, any analysis of fish abundance from electrofishing survey data is severely hampered by problems with gear and sampler selectivity, typically biased to over-represent large fish versus smaller fish. In the 2008/2009 NRSA data, across all surveys, over 300,000 fish were caught whose mean species weight (according to Barber et al. (2015)) is under 10 g, about half that number of fish were caught whose mean species weight is between 10 and 100 g, and about 100,000 fish were caught whose mean species weight is greater than 100 g. Given the gear and sampler-selectivity bias for large fish, true population numbers are likely skewed more heavily towards smaller fish than these data indicate.

**Fig. 8.**
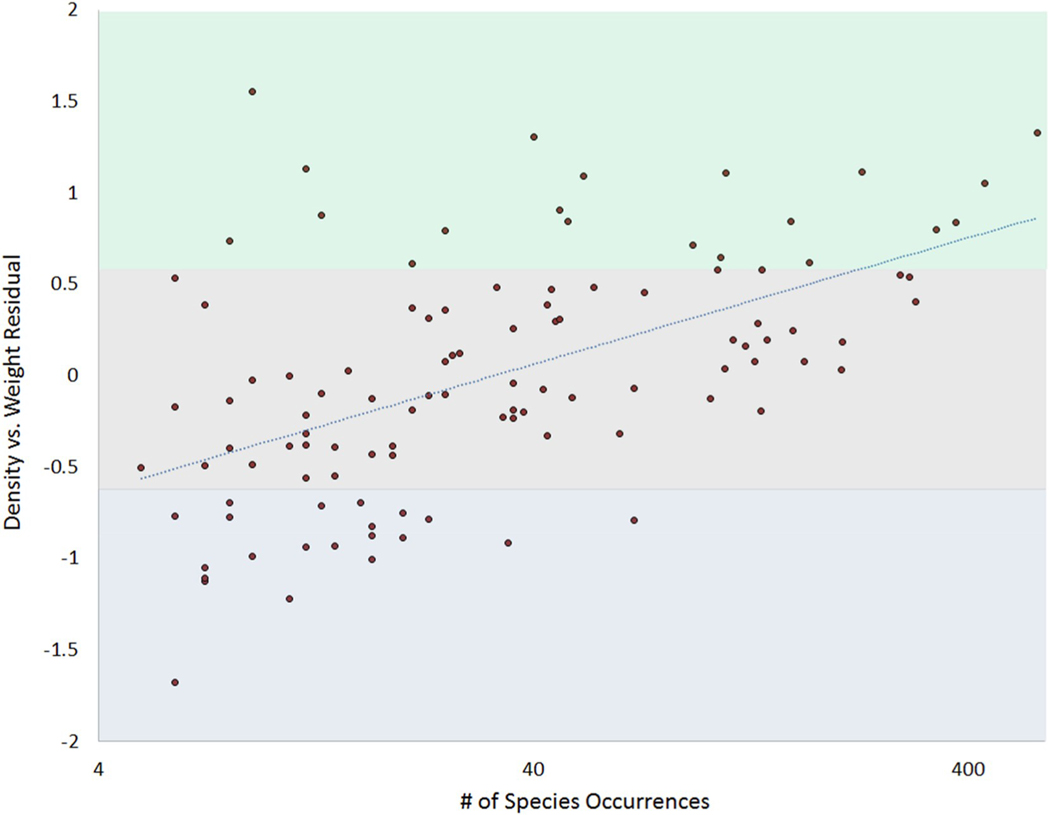
Plot of the residuals of Fig. 7 (Species Mean Density vs. Species Mean Weight) versus the number of surveys each species was found in (x-axis on a log10 scale, data from Barber et al. (2015). The green zone denotes positive residuals above one standard deviation (SD = 0.65) of the mean residual (zero by definition). The blue zone denotes residuals smaller than one standard deviation below the mean; the grey zone denotes residuals within one standard deviation of the mean.

Interestingly, we also found a positive relationship (Fig. 8) between the number of surveys that a species was found in and its residual in Fig. 7. More commonly-found species generally existed at greater densities than their size would suggest, while less common species existed at lower densities than their size would predict. When examining similarly-sized species within the NRSA data, we also found a positive correlation between the number of surveys a species occurred in and it’s mean density within those surveys.

Based on these findings, we modified the β values for these 108 species:

Equation 5b $$\beta^{*}=\beta -\Delta$$

Equation 6 $$\text{Δ}=0.15*\frac{\varepsilon }{3^{*}\sigma }$$

where ε is the species-specific residual in Fig. 8 and σ is the standard deviation of the residuals (σ = 0.65 for this dataset). Thus, Δ is ±0.15 for ε that are ±3 standard deviations from the mean, ± 0.1 units for residuals that are ±2 standard deviations from the mean, and ±0.05 for ε that are ±1 standard deviation from the mean. Since β ranges from 0.5 to 0.75, we deemed the scale of this adjustment to be an appropriate magnitude. Note that subtraction of Δ from β ensures that species with a positive ϵ in Fig. 8 have β* < β, leading to a higher relative abundance in a community.

In the future, if similar analyses can be done for other species and datasets, we could add Δ values for more species to the PiSCES database. A complex variety of factors interact to affect the density of a species in each system (physiological and morphological properties of the species itself, habitat suitability, ecological interactions, etc.). The raw data on species densities across sites looks much less organized than the mean value plot shown in Fig. 7. We don’t try to distill the importance of these various factors onto Δ; rather we target a mean species response across all systems where it occurs and assume a complex interaction of factors drives the deviation from the trend line in Fig. 8 (i.e., the residual for that species).

### 3.5. Species distribution characteristics

Based on our development of empirical occurrence models for the most commonly found species in the large survey database described in Section 3.3, we calculated four species characteristics describing how often and where species were found. These characteristics can be used to filter the PiSCES species database on the Database Explorer page (Fig. 5).

#### Ubiquity:

100*(# of surveys a species was found)/(# of surveys conducted within 8-digit HUCs where the species is known to occur); a percentage measure of how easily a species can be found within its known geographic extent; calculated for all species found in the survey database (n = 564); Ubiquity ranged from 0.093 to 74.4.

#### Extent:

100*(the number of 8-digit HUCs a species occurs in)/2200; a percentage measure of the geographic extent of a species; 2200 is an estimate of the total number of HUCs in the contiguous U.S.; Extent ranged 0.05 to 70.0 for the species in the PiSCES database with known distributions within the contiguous U.S. (n = 993).

#### Tolerance:

Percent of predicted occurrences (using the XGBoost models) within 10,000 streams with randomly-generated values of watershed area, elevation, slope, IWI, and BMMI; we used the Cholesky algorithm within the *scipy. linalg* library in Python (https://docs.scipy.org/doc/scipy-0.14.0/reference/linalg.html#module-scipy.linalg) to generate 10,000 random sets of these 5 parameters with a variance/covariance matrix equivalent to the empirical data; Tolerance is a measure of the diversity of environmental conditions a species is predicted to endure, independent of its geographical distribution; Tolerance varied from 0.02 to 93.9 for the 449 species with probabilistic occurrence models. Two species (Tallapoosa Sculpin and Tallapoosa Darter) with the highest Tolerance values were likely overestimated because they had a very small Extent, occurring in only a few HUCs, but were easily found in those HUCs (high Ubiquity). As a result, the probabilistic occurrence models for these species were insensitive to the stream characteristics at the survey sites, i.e., they predict a high occurrence probability for these species across a wide range of stream characteristics, even though many of those combinations of stream characteristics are well outside the range of characteristics of streams where these species are found. When it comes to predicting the occurrence of species at actual stream sites using the Assemblage Predictor, however, PiSCES only examines species that are known to occur within the HUC8 that the stream in question resides in, so overestimation of occurrence probabilities for species with small Extent should not be an issue.

#### Robustness:

a metric that combines Ubiquity, Extent, and Tolerance; calculated as (Extent^0.33^)*(Tolerance^0.33^)*(Ubiquity^0.33^); Robustness highlights species that occur in many HUCs, across many different stream conditions, and are typically seen in fish surveys within these HUCs; if any of these three conditions aren’t met, the Robustness of that species will be reduced; Robustness ranged from 0.56 to 46.3 for the 449 species with all three values necessary to calculate it. The Robustness of Tallapoosa Sculpin and Tallapoosa Darter were only average due to their minimal Extent, even though their Ubiquity and Tolerance were quite high.

### 3.6. Tribe

On the “Database Explorer,” the user can filter the PiSCES database to show groups of species (“tribes”) that share evolutionary commonality (a tribe is a taxonomic rank above genus but below family).

### 3.7. Ancillary characteristics

The PiSCES database also has the following information for each species:

- Origin: Native to US or Introduced
- Beneficial Use: Sport Fish, Non-Game, Subsistence
- Typical Systems Occupied: Caves, Springs, Headwaters, Creeks, Small Rivers, Medium River, Large Rivers, Lakes/Impoundments/Ponds/Canals/Ditches, Swamp/Marsh/Bayou, Coastal/Ocean
- Preferred Lotic Habitat: Riffles, Runs/Flowing Pools, Pools/Backwaters
- Preferred Location within the System: Benthic, Surface, Nearshore/Littoral, Pelagic
- Preferred Substrate: Mud/Silt/Detritus, Sand, Gravel, Rocks/Rubble/Boulders, Vegetation, Woody Debris/Brush
- Other Preferred Water Characteristics: Clear, Turbid, Warm, Cool, Cold, Lowland (low gradient), Upland (high gradient)

These descriptors were taken from the Peterson Guide, NatureServe.com and FishBase.org. Information on subsistence species was found in Kappen, Allison (Kappen et al., 2012). For most fish groups, species whose maximum body size was over 25 cm were considered sport fishes unless their rarity measure was 7 or greater. For Salmonids, this threshold was 20 cm; for Sunfish and Black Bass, the threshold was 15 cm. Species under these thresholds were designated as non-game.

The finalized PiSCES database contains information on 1018 fish species representing 202 genera. Table 2 shows categorization of the 48 tribes into sport fishes and non-game fishes (some tribes have members in each of these classes, but the tribe was defined based on most of its members), subsistence species (these tribes can be sportfish or non-game) and those entirely exotic to the contiguous U.S.

## 4. Validation

There are three components of PiSCES that interact to determine the community estimate for a stream:

- HUC8 fish distribution information
- Probabilistic Occurrence Models
- Community abundance

### 4.1. HUC8 fish distributions

The HUC8s where a species can be found within the United States are based on data provided by USGS and NatureServe, along with the distributional polygons in the Peterson Field Guide for several species, as explained earlier. However, there may be errors of omission (fish not listed in HUCs where they are present) and commission (fish listed in HUCs where they are not present) in our database. One of the advantages of PiSCES as an evolving web-based service is that this database is easily updated to rectify discovered errors, and these changes are then instantly available to PiSCES users.

To address errors of omission, we compiled a dataset of actual survey information from the databases delineated in Section 3.3. It was not critical that each database be completely independent of the others, as duplicate records (specific species/HUC combinations) were easily filtered out. As stated earlier, the compiled database had almost 40,000 unique species/HUC combinations representing 564 species. When compared to the existing PiSCES database (about 92,000 unique species/HUC records), we identified almost 7400 errors of omission, and the database was subsequently updated. Errors of commission are not possible to address using survey data but must rely on expert knowledge of local fish biodiversity. However, if these types of errors are brought to our attention, the database can be easily modified.

### 4.2. Occurrence modeling

Comparisons of modeled fish communities with actual survey samples often suffer from shortcomings (gear/sampler bias, seasonal movements, stochastic weather events, etc.) that introduce temporal and spatial variability into survey data (Freeman et al., 1988; Grossman et al., 1990; Mihaljevic et al., 2015; Falcy et al., 2016). However, using the parameters of watershed area, mean elevation, stream slope, IWI and BMMI to derive probabilistic occurrence models would hopefully allow estimation of a more plausible fish community.

The species-specific XGBoost models of species occurrence were tested against a validation dataset of almost 5000 site surveys. The confusion matrices below (Table 3) summarize results across the 432 species for which training-data occurrence models were calculated. These values were obtained using two different species-specific probability thresholds to determine whether the predicted probability of occurrence would be tabulated as a presence or absence for that species.

Both thresholds were calculated using modeling results on the training data. The table on the left (3a) used the average modeled probability of survey sites where the species was present (P_1_) and absent (P_0_). For example, for Species X, if P_1_ was 0.55 and P_0_ was 0.23, the threshold probability would be (0.55 + 0.23)/2 = 0.39. The table on the right (3b) used the maximum value of (P_1_ – SD(P_1_)) and P_0_, where SD (P_1_) was the standard deviation of modeled probabilities for sites where the species was found. This threshold produces fewer false negatives (lower left cell), but more false positives (upper right cell). For a given application, it may be more important to accurately predict the presence of a species when it occurs, versus accurately predicting the absence of a species when it isn’t there. If this is the case, this second threshold would provide better results. The PiSCES interface will allow the user to choose from several thresholds for deciding species presence/absence based on predicted probabilities of occurrence.

Cohen’s Kappa is a statistic often recommended for measurement of model fit based on a confusion matrix (Manel et al., 2001; Fielding, 1999). The numbers in Table 3 produce a Kappa of 0.39 for the results on the left, and 0.32 for the data on the right, which indicates moderate model performance (Landis and Koch, 1977). Based on data on the left, the overall model accuracy for the testing sites was 82% (75% for the data on the right), the sensitivity (correct prediction of true positives) was 63% (74% for the right data), and the specificity (correct prediction of true negatives) was 85% (76% for the right data). This shows the tradeoffs of raising/lowering the decision threshold. Lowering the threshold, as resulted from the alternate threshold calculation for the data on the right, increased the model’s sensitivity, but decreased its specificity. Using this threshold would lower the chances of the model predicting the absence of a species when it occurs, while increasing the likelihood that the model predicts the presence of a species when it doesn’t occur.

### 4.3. Community abundance

The ecological concept that there should be many more small-bodied organisms in a community at the base of the food chain, and lesser numbers of larger species higher on the food chain, is robust and well-supported (see references to the negative relationship of body size to abundance in a community given earlier). Even so, the calculator in PiSCES that implements this concept using thinning coefficients and mean body size of all the species in a community is admittedly an oversimplification. The size spectrum is meant to give the user a sense of relative abundance within a community that exhibits body-size diversity. In communities where most species have nearly the same body size (a mixture of various minnows and darters, for example), species’ relative abundance could be highly variable from stream to stream depending on competitive interactions and stochastic historic events.

## 5. Conclusion

In summarizing PiSCES development outcomes and the introductory discussion on motivation, design and intended use, three major benefits are derived from its final design and functionality. In stand-alone mode, PiSCES allows users to develop hypothesized fish communities based on known distributions in lotic systems across the US. This functionality has numerous applications to serve a multitude of current assessment programs and research endeavors. Secondly, within an integrated environmental modeling framework (Johnston et al., 2011, 2017), PiSCES provides a service necessary to perform hydroecological assessments which link mechanistic hydrology models with ecological models to achieve prediction goals. Finally, PiSCES′ general flexibility allows users to modify the “best estimate” of a fish community based on additional waterbody-specific data. This functionality, established as an important design requirement, enhances the capabilities of both standalone use and integrated modeling applications for which it was created.

## 6. Availability

PiSCES is freely available on the web at: https://qed.epacdx.net/pisces/

## Supplementary Material

sup1

## Figures and Tables

**Fig. 6a.**
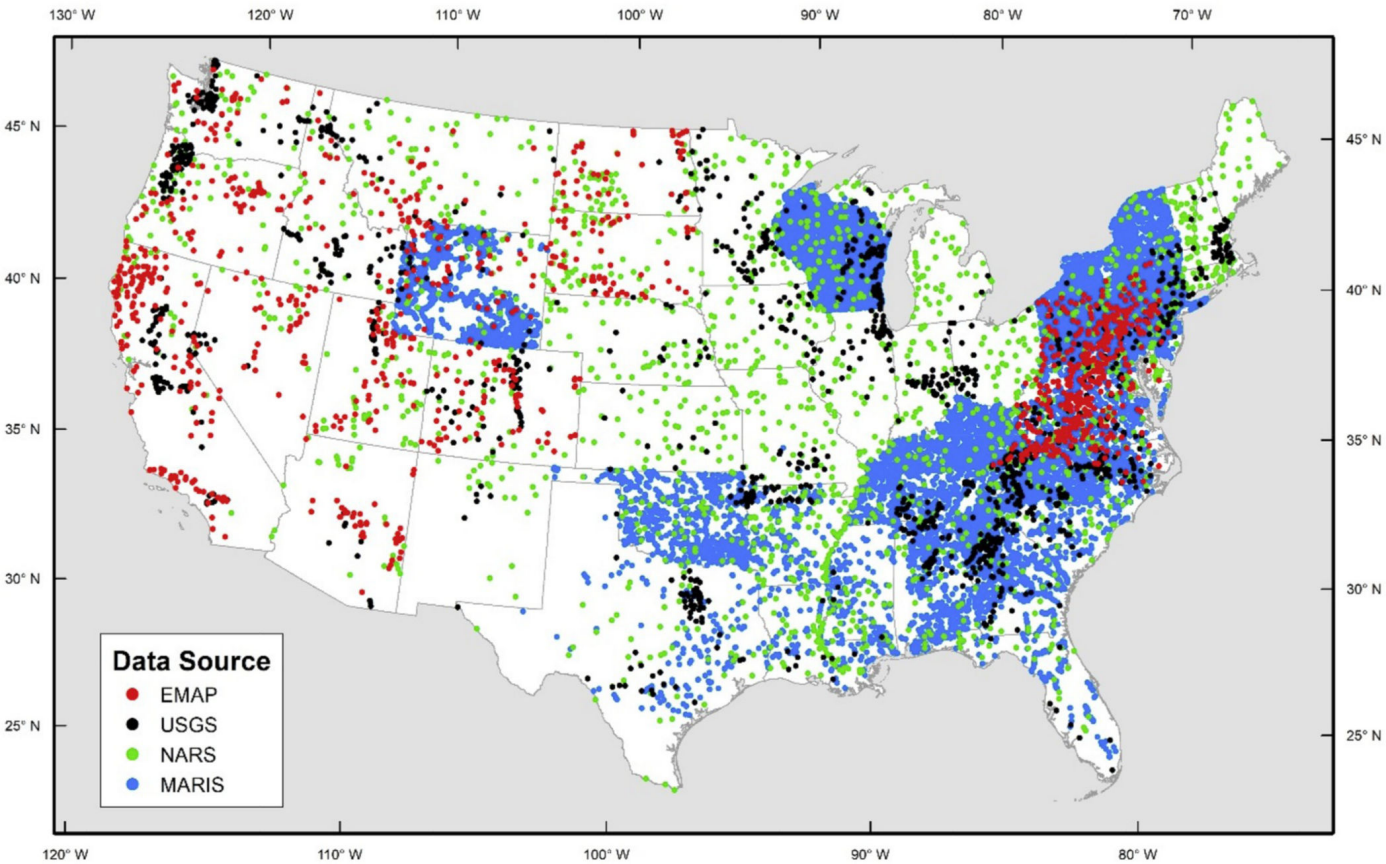
Map of fish survey locations within the contiguous US from four different data sources.

**Fig. 6b.**
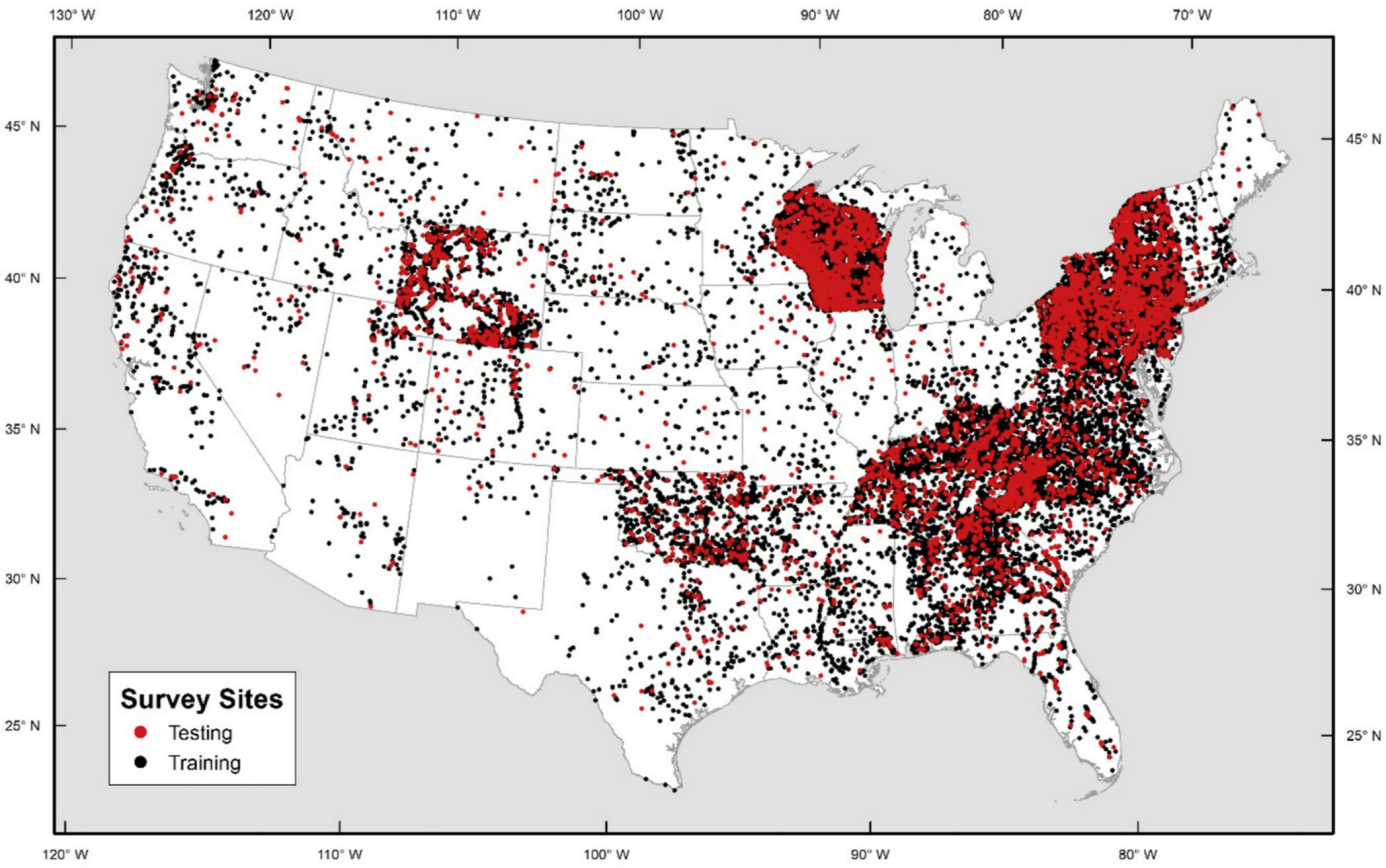
Distribution of training sites (black dots) and testing sites (red dots) within the fish survey data.

**Table 1:** Calculations for determining a hypothetical community abundance and biomass spectrum. Total abundance is 10,000 fish.

Mean Weight (g)	Thinning Coefficient	Abundance	Relative Abundance	N	Biomass (g)

0.1	0.50	3.16	0.534	3803	380
0.3	0.50	1.83	0.309	2196	659
5	0.55	0.41	0.070	496	2479
8	0.57	0.31	0.052	370	2963
25	0.61	0.14	0.024	171	4268
120	0.67	0.04	0.007	50	5940
200	0.69	0.03	0.004	32	6311

**Table 2:** Categorization of the 48 fish tribes in PiSCES.

Sportfish	Non-Game	Subsistence	Exclusively Exotic

Black Bass	Anchovy	Anchovy	Carp
Bowfin	Cavefish	Bowfin	Cichlid (Exception: Rio Grande Cichlid)
Burbot	Darter	Burbot	Knifefish
Carp	Goby	Catfish	Loach
Catfish	Knifefish	Cod	Snakehead
Cichlid	Lamprey	Drum	Tetra
Cod	Livebearer	Eel	
Drum	Loach	Gar	
Eel	Madtom	Lamprey	
Flounder	Minnow	Minnow	
Gar	Mudminnow	Mullet	
Paddlefish	Mullet	Paddlefish	
Perch	Pipefish	Perch	
Pike	Pupfish	Pike	
Salmon/Trout	Pygmy Sunfish	Salmon/Trout	
Shad/Herring	Sculpin	Shad/Herring	
Skipjack	Silverside	Smelt	
Snakehead	Smelt	Sturgeon	
Stingray	Splitfin	Sucker	
Sturgeon	Stickleback	Trout-Perch	
Sucker	Surfperch	White Bass	
Sunfish	Tetra	Whitefish	
White Bass	Topminnow		
Whitefish	Trout-Perch		

**Table 3:** Confusion matrix of predicted outcomes within the testing survey sites compared to actual presence/absence of the 432 species found in at least 25 surveys within the training data. See text for a definition of the thresholds used to compute the left (3a) and right (3b) tables.

		Surveys				Surveys	
		Present	Absent			Present	Absent

Predictions	Present	27333	40778	Predictions	Present	32244	67394
	Absent	15985	235264		Absent	11074	208648
	**Threshold: Average(P_0_, P_1_)**				**Threshold: Max(P_0_, P_1_-StDev(P_1_))**		
