# Supplementary material for: PiSCES: Pi(scine) stream community estimation system
Source: Environ Model Softw. Author manuscript; Available in PMC 2021 May 1. (PMC7970533; doi:10.1016/j.envsoft.2020.104703)
Supplement: sup1 [file NIHMS1603505-supplement-sup1.docx]

**Research data for this article can be found at:**

**Cyterski, M. (2020). PiSCES Dataset [Data set]. U.S. EPA Office of Research and Development (ORD).** [**https://doi.org/10.23719/1518775**](https://doi.org/10.23719/1518775)
